# Gene Expression Profiling of Mono- and Co-Culture Models of the Respiratory Tract Exposed to Crystalline Quartz under Submerged and Air-Liquid Interface Conditions

**DOI:** 10.3390/ijms23147773

**Published:** 2022-07-14

**Authors:** Alexandra Friesen, Susanne Fritsch-Decker, Matthias Hufnagel, Sonja Mülhopt, Dieter Stapf, Carsten Weiss, Andrea Hartwig

**Affiliations:** 1 Department of Food Chemistry and Toxicology, Institute of Applied Biosciences (IAB), Karlsruhe Institute of Technology (KIT), 76131 Karlsruhe, Germany; alexandra.friesen@kit.edu (A.F.); matthias.hufnagel@gmail.com (M.H.); 2 Institute of Biological and Chemical Systems, Biological Information Processing, Karlsruhe Institute of Technology (KIT), 76344 Eggenstein-Leopoldshafen, Germany; susanne.fritsch-decker@kit.edu (S.F.-D.); sonja.muelhopt@kit.edu (S.M.); dieter.stapf@kit.edu (D.S.)

**Keywords:** quartz, pulmonary toxicity, air-liquid interface, co-culture, transcriptional toxicity profile, gene expression, particle

## Abstract

In vitro lung cell models like air-liquid interface (ALI) and 3D cell cultures have advanced greatly in recent years, being especially valuable for testing advanced materials (e.g., nanomaterials, fibrous substances) when considering inhalative exposure. Within this study, we established submerged and ALI cell culture models utilizing A549 cells as mono-cultures and co-cultures with differentiated THP-1 (dTHP-1), as well as mono-cultures of dTHP-1. After ALI and submerged exposures towards α-quartz particles (Min-U-Sil5), with depositions ranging from 15 to 60 µg/cm^2^, comparison was made with respect to their transcriptional cellular responses employing high-throughput RT-qPCR. A significant dose- and time-dependent induction of genes coding for inflammatory proteins, e.g., *IL-1A*, *IL-1B*, *IL-6*, *IL-8*, and *CCL22*, as well as genes associated with oxidative stress response such as *SOD2*, was observed, even more pronounced in co-cultures. Changes in the expression of similar genes were more pronounced under submerged conditions when compared to ALI exposure in the case of A549 mono-cultures. Hereby, the activation of the NF-κB signaling pathway and the NLRP3 inflammasome seem to play an important role. Regarding genotoxicity, neither DNA strand breaks in ALI cultivated cells nor a transcriptional response to DNA damage were observed. Altogether, the toxicological responses depended considerably on the cell culture model and exposure scenario, relevant to be considered to improve toxicological risk assessment.

## 1. Introduction

Crystalline quartz or α-quartz is found ubiquitously in rock and soil, and especially occupational inhalative exposure towards respective dusts at workplaces pose a problem in different sectors in industry [1]. Inhalable dusts containing crystalline quartz particles have been classified as carcinogenic to humans (group 1) by IARC since 1997 [2]. Apart from lung cancer, elevated levels of quartz increase the risk of silicosis, one of the most studied and known occupational diseases [3]. Critical target cells in the lung are macrophages, neutrophils, epithelial cells and fibroblasts which are linked to pathogenic processes leading to inflammation and fibrosis [4].

To assess the toxicity of quartz particles, many studies, including in vivo and in vitro testing, have already been performed. However, to implement the rising demand of reducing animal testing in the context of the 3 Rs (replace, reduce, refine), there is a requirement to improve current in vitro models and to establish more physiological conditions than in classical submerged exposure experiments. This led to the development of various advanced models, such as cells grown at an air-liquid interface (ALI) to simulate conditions in the lung more realistically, and co-culture models employing different cell types allowing to analyze interactions between the cells or sophisticated organ-on-a-chip-models [5,6,7]. Nevertheless, most studies performed with quartz particles employed submerged approaches and led to contradictory results. Especially studies utilizing an ALI approach for quartz exposure are scarce. In a study by Barosova et al., a 3D model composed of A549 (lung epithelial -), THP-1 (macrophage like -), and MRC-5 (fibroblast -) cells was applied to assess the toxicity of the two reference quartz particle types, Min-U-Sil5 and Dörentrup Quartz 12 (DQ12) [8]. In another study by Öhlinger et al., ALI and submerged exposure of A549 cells with Min-U-Sil5 was compared, but only with regard to cytotoxicity [9]. Most other studies exclusively employed submerged exposure. Studies applying co-cultures are limited as well, the most recent investigations used a 3D transwell model composed of A549, THP-1 and endothelial cells (Ea.hy926 or HIVE-26, respectively) or primary rat cells, all exposed to Min-U-Sil5 [10,11,12]. Conditioned media approaches with medium transfer between different cells after exposure to quartz have also been reported [13,14].

The underlying mechanism of quartz toxicity is still not completely understood. Several physicochemical properties of quartz might contribute to its toxicity: reactive silanol groups at the surface, direct ROS generation due to fracturing of silanol bonds during e.g., grinding and drilling, or trace contaminants such as redox-active iron [4]. All of these factors promote the formation of hydroxyl radicals and superoxide anions. In turn, these radicals interact with various cellular constituents, such as proteins, cellular membranes, and DNA [15,16]. Quartz has been reported to induce oxidative stress and DNA damage in A549 and RLE-6TN lung epithelial cells [17,18,19], as well as oxidative stress in BEAS-2B bronchial cells [20]. Since quartz has also been known to induce inflammation, the involvement of the Nuclear Factor-κB (NF-κB) pathway has been discussed and examined in vitro, exposing RLE-6TN cells, as well as in vivo in rats [14,21,22]. Furthermore, the involvement of the NLRP3 (NOD-, LRR- and pyrin domain containing protein 3) inflammasome was proposed as a possible mechanism in bronchial epithelial cell lines 16HBE14o-, BEAS-2B, and NHBE, as well as in THP-1 cells [23,24,25]. Moreover, pro-fibrotic changes were reported in lung fibroblasts alone and in a conditioned media setting with macrophages [13,26]. Notably, all these mechanistic studies concerning quartz toxicity have been performed under submerged exposure conditions.

Within a recent study, we compared in vitro models based on the human alveolar epithelial cell line A549 in mono- as well as co-cultures with macrophage-like differentiated human THP-1 cells (dTHP-1) grown under either submerged or ALI conditions upon exposure towards Min-U-Sil5, a widely used and well-studied type of natural crystalline quartz. Especially, we investigated cytotoxicity and inflammatory cytokine release at different concentrations, demonstrating that cells cultivated at an ALI exhibit a lower sensitivity against quartz particles. The particles used for this study were characterized via dynamic light scattering (DLS) und transmission electron microscopy (TEM), which revealed a broad size distribution, peaking at a diameter of 0.95 µm. Its purity of 99.4% and high crystallinity qualified Min-U-Sil5 for mechanistic toxicity studies as well [27]. Within the present study, we aimed to elucidate underlying molecular mechanisms and signaling pathways triggered by quartz, utilizing the same quartz particles. We adapted and applied a high-throughput RT-qPCR (HT RT-qPCR) approach, which has been established in our laboratory and successfully applied to assess toxicity profiles upon treatment with different (nano)materials [28]. The method allows the simultaneous assessment of the expression of 95 genes involved in key toxicological pathways related to inflammation, genotoxicity, redox and stress signaling as well as fibrosis in 96 samples. Qualitative and quantitative differences of the response between the various cell culture models were evaluated and put into a broader toxicological context relevant for hazard assessment of particulate matter in general and quartz in particular. 

## 2. Results

To gain a deeper insight into the transcriptional toxicity profile of quartz particles, the different cell culture models were assessed via HT RT-qPCR after exposure to three doses of the α-quartz Min-U-Sil5. ALI A549 mono- and A549/dTHP-1 co-cultures were exposed to 15, 30, or 60 µg/cm^2^ of quartz, with slight deviations in depositions as quantified in the exposure chamber. This dose range was chosen based on our own preliminary experiments, which demonstrated biologically relevant transcriptional changes starting from 15 µg/cm^2^ (data not shown) and based on published literature [20,29]. The doses for submerged exposures were chosen correspondingly; here, the total deposited doses were considered to comply with the applied nominal doses based on particle size and their rapid sedimentation, which has previously been described in other studies [30]. Regarding dTHP-1 cells, lower doses were applied due to the higher cytotoxicity when compared to A549 cells [27]. Furthermore, ALI exposure experiments were not performed with this cell line as these cells do not build a confluent monolayer. The regular post-incubation period for all cultures was 24 h, if not stated otherwise. For qPCR results, changes above a log_2_-value of 1 or below a log_2_-value of -1 (which corresponds to a doubling or halving of expression levels, respectively) are considered biologically relevant. The results are depicted as heatmaps, with a selection of noteworthy genes shown in more detail afterwards. Additional genotoxicity testing to detect DNA strand breaks was performed for the two ALI cultures via Alkaline Unwinding. 

### 2.1. Inflammation- and Oxidative Stress-Related Gene Expression Is Upregulated in A549 and dTHP-1 Mono-Cultures upon Exposure to Quartz

**Figure 1:**
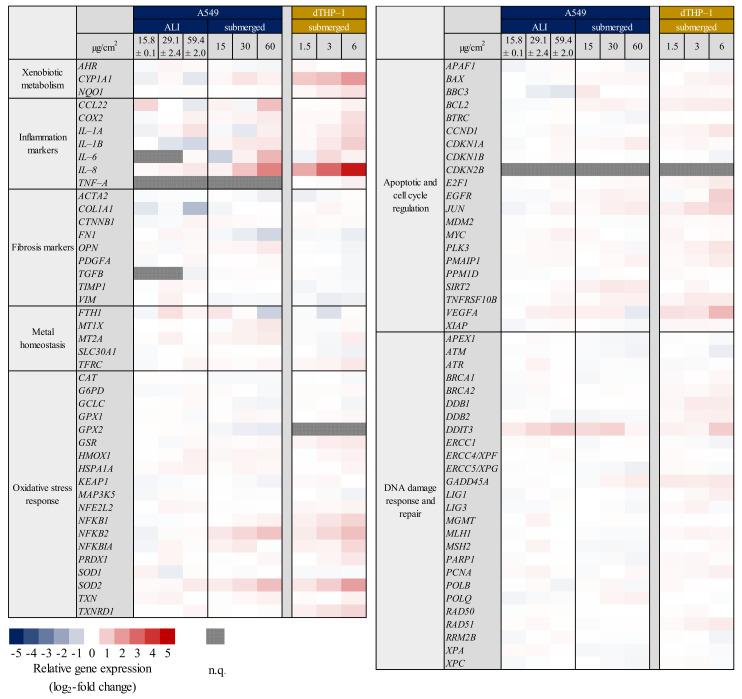
Gene expression profiles of A549 and dTHP-1 mono-cultures after air-liquid interface (ALI) and/or submerged exposure to Min-U-Sil5. A549 and dTHP-1 cells were exposed to three different doses of Min-U-Sil5 and incubated for 24 h. The results are depicted as the log_2_-fold change of the relative gene expression. A red color represents the induction, a blue color the repression of a gene. The means of at least three independent experiments are displayed. Applied doses in ALI exposures were calculated as means ± SD from three independent experiments. n.q.: not quantifiable due to low expression levels.

**Figure 2:**
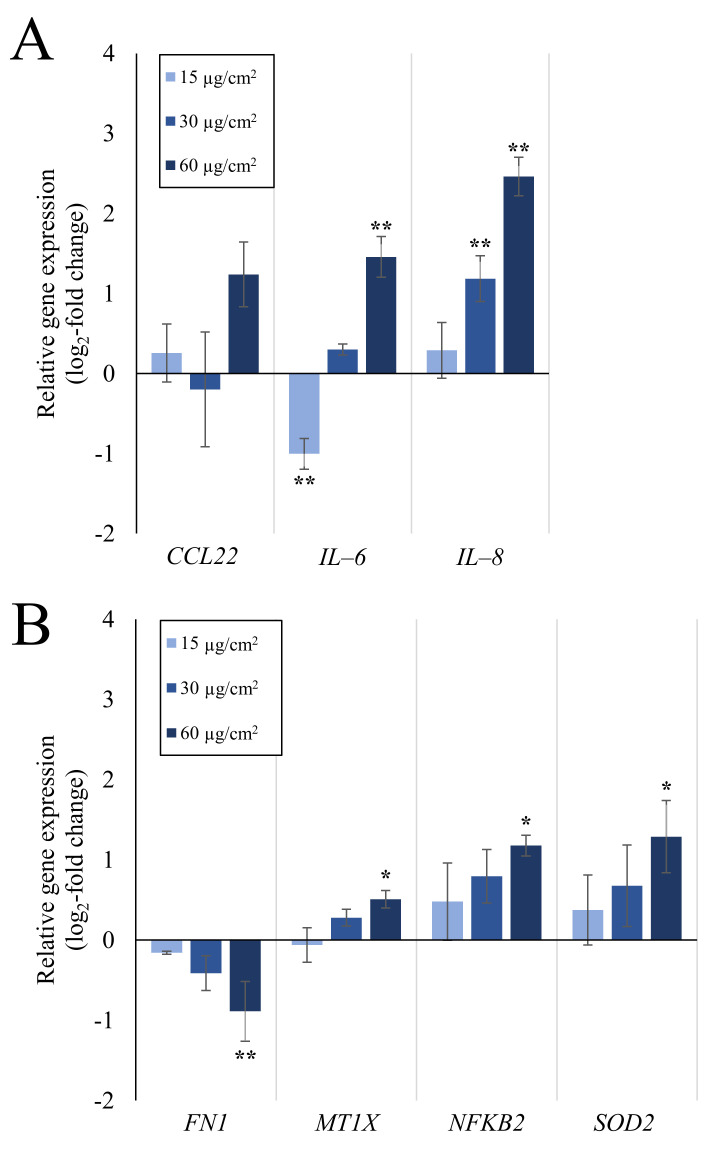
Impact of submerged Min-U-Sil5 exposure on the expression of inflammatory genes (**A**) and genes various gene clusters (**B**) significantly altered in their expression in A549 mono-cultures. A549 cells were exposed to three different doses of Min-U-Sil5 and incubated for 24 h. Results are depicted as the log_2_-fold change of the relative gene expression. The means ± SD of three independent experiments performed in duplicates are displayed. Statistical analysis was performed to assess differences between the exposed cells and the negative control with one-way ANOVA followed by Dunnett’s post hoc test: * (*p* ≤ 0.05), ** (*p* ≤ 0.01).

**Figure 3:**
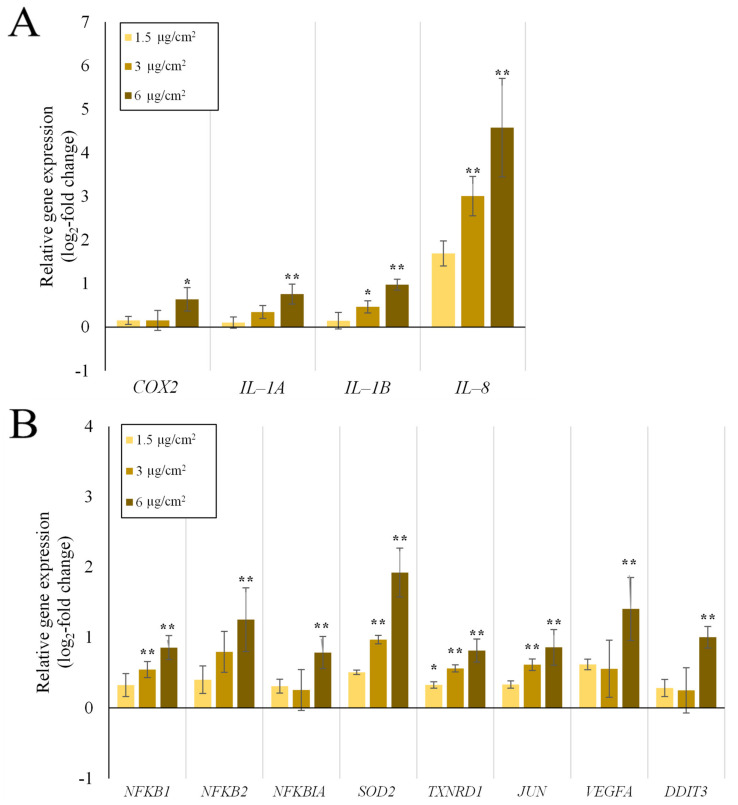
Impact of submerged Min-U-Sil5 exposure on the expression of inflammatory genes (**A**) and genes of various gene clusters (**B**) significantly altered in their expression in dTHP-1 mono-cultures. dTHP-1 cells were exposed to three different doses of Min-U-Sil5 and incubated for 24 h. Results are depicted as the log_2_-fold change of the relative gene expression. The means ± SD of three independent experiments performed in duplicates are displayed. Statistical analysis was performed to assess differences between the exposed cells and the negative control with one-way ANOVA followed by Dunnett’s post hoc test: * (*p* ≤ 0.05), ** (*p* ≤ 0.01).

Gene expression analysis of A549 mono-cultures after submerged and ALI exposure and of dTHP-1 cells after submerged exposures was performed via HT RT-qPCR. The results for the whole gene set are displayed in Figure 1 as a heatmap; a selection of significantly altered genes considered also to be relevant is shown in more detail in Figure 2 (A549) and Figure 3 (dTHP-1). The precise log_2_-values for all genes are depicted in Supplementary Table S1.

As evident in Figure 1, A549 cells exposed to quartz at the ALI did not display relevant changes in gene expression, with the exception of *DDIT3*, coding for a DNA damage inducible, pro-apoptotic transcription factor. In contrast, under submerged conditions, the exposure produced distinct concentration-dependent changes in gene expression. In case of A549 cells, the most significant changes were observed in the gene clusters “inflammation” and—less pronounced— “oxidative stress response”. Examples for upregulated genes are *CCL22, IL-6,* and *IL-8* as markers for inflammatory response, and *NFKB2, NFKBIA,* and *SOD2* related to the oxidative stress response. The highest changes were observed in the gene *IL-8*, which was induced 2.3-fold at the medium dose and 5.5-fold at the high dose. *IL-6* displayed a non-uniform behavior by being repressed 2-fold at the low dose and being induced 2.8-fold at the high dose. Additionally, the expression of single genes from other clusters was altered after submerged exposure, with the expression of *MT1X* from “metal homeostasis” and *FN1* from “fibrosis markers” being slightly down-regulated. 

The dTHP-1 mono-culture showed diverse genes being slightly induced throughout the whole gene set, with *IL-8* standing out with a highly concentration-dependent activation pattern and an induction of 32-fold at the highest concentration. Another prominent gene was *SOD2* with an induction of up to 4-fold. *CYP1A1* displayed an apparent induction in the heatmap, which can be neglected because of the high deviations between experiments. Statistically significant changes in other genes were already observed at the medium dose for *IL-1B, NFKB1, SOD2, TXNRD1,* and *JUN*. Nevertheless, biologically relevant activations above a log_2_ value of 1, which corresponds to a two-fold activation, were only observed at the higher dose of 6 µg/cm^2^. Deviations between experiments appear smaller than for A549 mono-culture experiments. Since the doses were reduced 10-fold for the dTHP-1 experiments when compared to A549 cells and the gene expression was still significantly elevated for these genes, dTHP-1 cells appear to be more sensitive towards Min-U-Sil5 than A549 cells. 

### 2.2. Inflammatory Gene Induction Is More Pronounced in A549/dTHP-1 Co-Cultures Compared to Mono-Cultures in Response to Quartz

**Figure 4:**
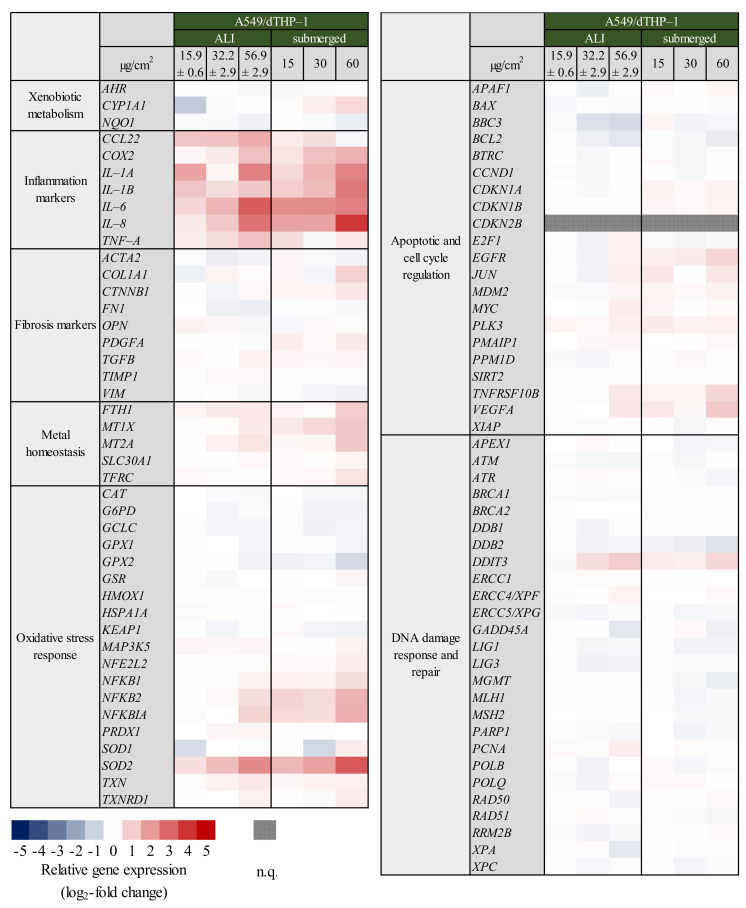
Gene expression profiles of A549/dTHP-1 co-cultures after air-liquid interface (ALI) and submerged exposure to Min-U-Sil5. Co-cultures comprised of A549 and dTHP-1 cells were exposed to three different doses of Min-U-Sil5 and incubated for 24 h. The results are depicted as the log_2_-fold change of the relative gene expression. A red color represents the induction, a blue color is the repression of a gene. The means of at least three independent experiments are displayed. Applied doses in ALI exposures were calculated as means ± SD from three independent experiments. n.q.: not quantifiable due to low expression levels.

**Figure 5:**
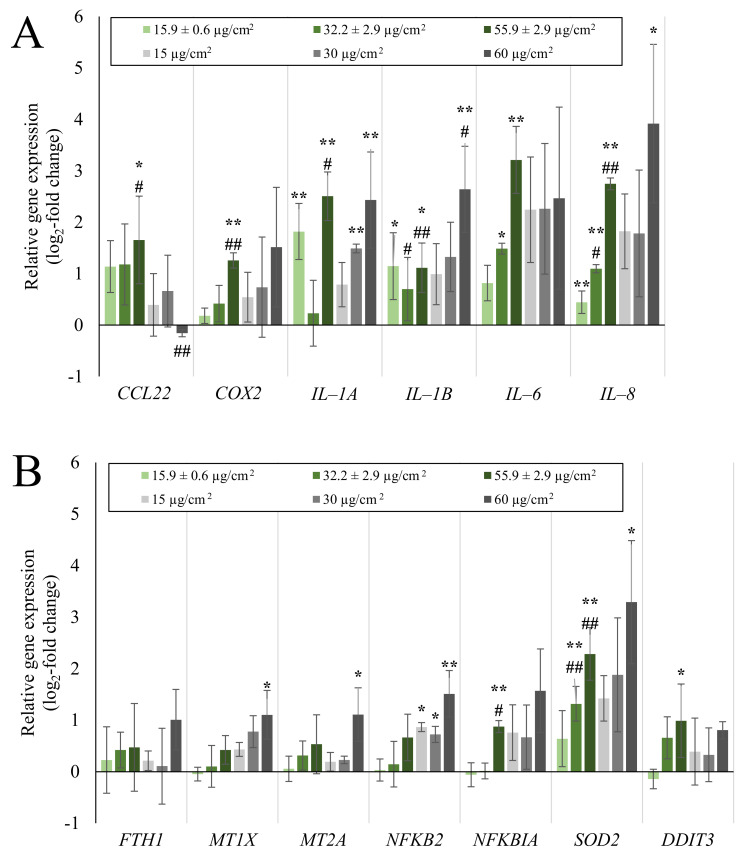
Impact of Min-U-Sil5 exposure on the expression of inflammatory genes (**A**) and genes of various gene clusters (**B**) significantly altered in their expression in pulmonary co-cultures after ALI (green bars) or submerged exposure (grey bars). Co-cultures comprised of A549 and dTHP-1 cells were exposed to three different doses of Min-U-Sil5 and incubated for 24 h. Results are depicted as the log_2_-fold change of the relative gene expression. The means ± SD of three independent experiments performed in duplicates are displayed. Statistical analysis was performed to assess differences between the exposed cells and the negative control with one-way ANOVA followed by Dunnett’s post hoc test: * (*p* ≤ 0.05), ** (*p* ≤ 0.01), or to compare differences between the A549 mono- and A549/dTHP-1 co-culture with student’s *t*-test: # (*p* ≤ 0.05), ## (*p* ≤ 0.01).

For the construction of co-cultures, dTHP-1 cells were seeded on top of A549 cells and exposed to quartz analogous to the mono-culture experiments. The results for the whole gene set are displayed in Figure 4 as a heatmap; a selection of relevant genes is shown in more detail in Figure 5. The precise log_2_-values for all genes are depicted in Supplementary Table S2.

Perhaps most evident from the overview presented in Figure 4, under co-culture conditions as opposed to mono-culture conditions shown in Figure 1, especially genes related to the inflammatory response were induced far more pronounced under submerged, but also under ALI conditions. In principle, similar genes as observed in the A549 and dTHP-1 mono-cultures were activated in the co-culture. Regarding ALI exposures, a concentration-dependent induction in inflammatory gene expression was noted, the exceptions being *IL-1A* and *IL-1B.* In case of *CCL22, COX2, IL-6*, and *IL-8* the highest dose of 56.9 ± 2.90 µg/cm^2^ induced a statistically significant increase in gene expression. Comparing these results to the gene expression profiles of mono-cultures at the ALI, the observed effects are especially pronounced. Moreover, expression of some specific genes such as *IL-6* and *TNF-A* could not be detected in the A549 mono-culture at all. This implies a synergistic interaction between dTHP-1 and A549 cells in the response to quartz exposure. Furthermore, similar effects were observed under submerged conditions. Inflammatory gene expression was mostly enhanced by the addition of dTHP-1 cells, especially for *COX2*, *IL-1A*, and *IL-8*, which could be related to the stronger activation of these genes already observed in the A549 mono-culture. The general effect of increased induction after submerged rather than ALI exposure was observed in the co-culture as well, albeit less pronounced and with a few notable exceptions. *CCL22* and *TNF-A*, as well as *IL-1A* did not follow this pattern: the induction at the ALI appeared stronger than or similar to the effects after submerged exposure, respectively. 

Regarding the cluster “oxidative stress response”, the gene encoding the superoxide dismutase 2 (*SOD2*), which is dependent on the NF-κB signaling pathway [31], showed a significant up-regulation at the middle and highest dose and a significant difference to the mono-culture. In contrast, the addition of dTHP-1 cells did not result in a significantly stronger up-regulation for most of the NF-κB related genes (*NFKB1, NFKB2,* and *NFKBIA)*, neither under submerged conditions nor at the ALI, with the exception of *NFKBIA* at the highest dose. 

Similar to the mono-culture, there was a series of various genes from different gene clusters affected as well, namely *MT1X*, *MT2A, JUN,* and *VEGFA* being slightly activated. The apoptotic mediator *TNFRSF10B* also showed a slight induction in the submerged co-culture setting. Contrarily, while a significant induction occurred at the highest quartz dose after ALI exposure, there was no change in the expression of DNA damage response mediator *DDIT3* compared to the mono-culture.

### 2.3. Absence of DNA Strand Breaks after ALI Exposure to Quartz

**Figure 6:**
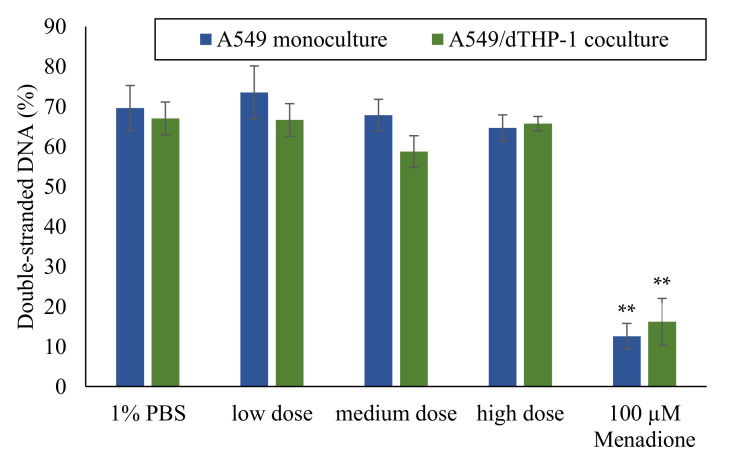
Assessment of DNA strand breaks after ALI-exposure of A549 mono-cultures and A549/dTHP-1 co-cultures to Min-U-Sil5. A549 and dTHP-1 cells were exposed to three different doses (µg/cm^2^) of Min-U-Sil5 (A549: 15.8 ± 0.8 (low), 29.1 ± 2.4 (medium), 59.4 ± 2.0 (high); A549/dTHP-1: 15.9 ± 0.6 (low): 32.2 ± 2.9 (medium): 55.9 ± 2.9 (high) and incubated for 24 h. As a positive control, cells were treated with menadione to trigger oxidative DNA damage. The means of three independent experiments are displayed. Applied doses were calculated as means ± SD from three independent experiments. Statistical analysis was performed to assess differences between the quartz exposed cells and the PBS control applying the student’s *t*-test: ** (*p* ≤ 0.01).

As shown in Figure 1 and Figure 4, no relevant changes were observed with regard to genes related to DNA damage signaling, especially *GADD45A*. Changes related to any specific DNA repair pathways were also not evident, suggesting the absence of DNA damage upon treatment with Min-U-Sil5, both in mono- or co-cultures under submerged or ALI conditions. This was followed up by a second approach, namely the quantification of DNA strand breaks via Alkaline Unwinding. Experiments were performed in A549 mono-cultures and A549/dTHP-1 co-cultures exposed to Min-U-Sil5 at the ALI. Within this test system, an increase of DNA strand breaks would result in a decrease in the percentage of double-stranded DNA [32]. As shown in Figure 6, no significant alterations in the degree of double-stranded DNA were observed after treatment with Min-U-Sil5 in both mono- or co-cultures at the ALI when compared to the PBS-treated negative control, indicating the absence of DNA strand breaks. 

### 2.4. Expression of Inflammatory and Oxidative Stress Response Genes Induced by Quartz Is Highly Time-Dependent

**Figure 7:**
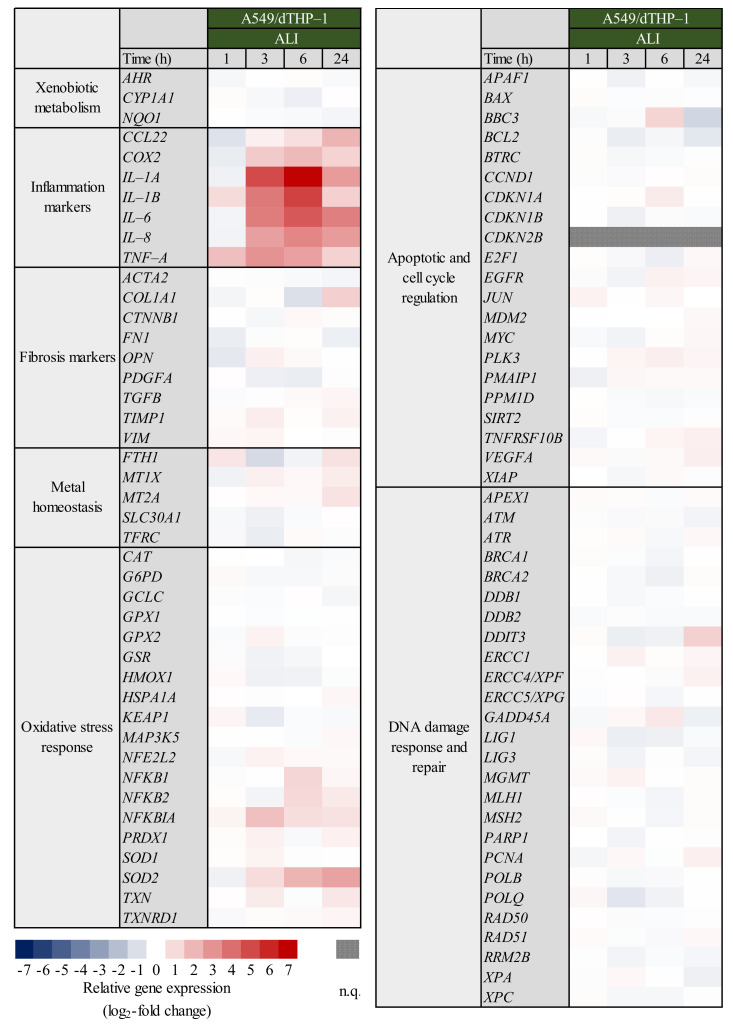
Impact of post-incubation time on Min-U-Sil5 mediated gene expression in A549/dTHP-1 co-cultures after ALI exposure. Co-cultures comprised of A549 and dTHP-1 cells were exposed to 56.9 ± 2.94 µg/cm^2^ of Min-U-Sil5 and incubated for 1, 3, 6 or 24 h. The results are depicted as the log_2_-fold change of the relative gene expression. A red color represents the induction, a blue color the repression of a gene. The means of at least three independent experiments are displayed. Applied doses in ALI exposures were calculated as means ± SD from three independent experiments. n.q.: not quantifiable due to low expression levels.

**Figure 8:**
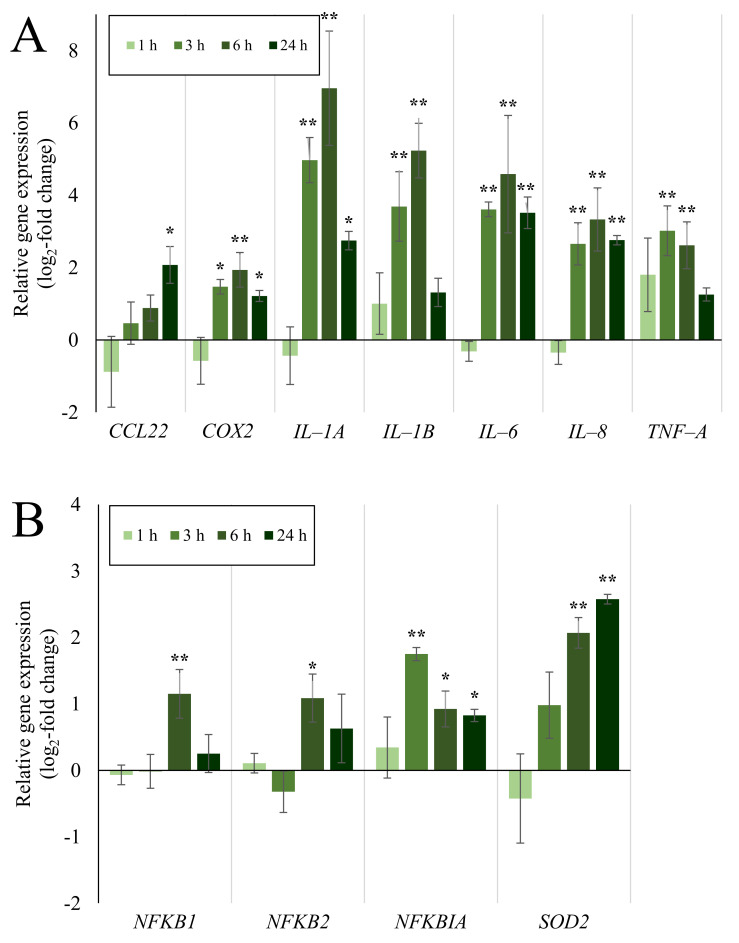
Impact of post-incubation time on Min-U-Sil5-mediated induction of inflammatory (**A**) and oxidative stress (**B**) genes in pulmonary co-cultures treated at the ALI. Co-cultures comprised of A549 and dTHP-1 cells were exposed to 56.9 ± 2.94 µg/cm^2^ of Min-U-Sil5 and incubated for 1, 3, 6 or 24 h. The results are depicted as the log_2_-fold change of the relative gene expression. The means ± SD of three independent experiments performed in duplicates are displayed. Statistical analysis was performed to assess differences between the exposed cells and the negative control with ANOVA-Dunnett’s: * (*p* ≤ 0.05), ** (*p* ≤ 0.01).

To investigate whether alterations in gene expression after Min-U-Sil5 exposure follows a time dependent pattern, A549/dTHP-1 co-cultures at the ALI were exposed to the highest dose of quartz particles (56.9 ± 2.94 µg/cm^2^), and changes in gene expression were assessed after 1, 3, 6 and 24 h post-incubation. A full heatmap with the whole gene set is available in Figure 7. The results for the most relevant genes are shown in Figure 8.

Inflammatory gene expression was highly time-dependent with a peak after a post-incubation time of six hours for most genes, i.e., *COX2, IL-1A, IL-1B, IL-6,* and *IL-8.* Especially *IL-1A* and *IL-1B,* which were only moderately activated after 24 h showed the highest increase in gene expression, reaching 125-fold and 38-fold levels, respectively, after six hours. In contrast to *IL-8*, which was identified as the gene with the highest induction after 24 h with a seven-fold induction, *IL-1* gene expression was far more pronounced at earlier time points. Some of the inflammatory genes deviated from the observed maximum at six hours: *TNF-A* was induced strongest after three hours, while *CCL22* showed a maximum at the latest time point of 24 h.

The expression of NF-κB related genes also followed a time dependency, with *NFKB1* and *NFKB2* peaking at six and *NFKBIA* at three hours, while *SOD2* reached its maximum after 24 h.

## 3. Discussion

In this study, we applied a co-culture model comprised of the alveolar epithelial cell line A549 and differentiated macrophage-like dTHP-1 cells to investigate the transcriptional toxicity profiles of quartz particles, following up on a recent report evaluating cytotoxicity and cytokine release after quartz exposure using the same cell culture models [27]. Within this study, it was our objective to assess potential differences between the co-culture model and the corresponding mono-cultures as well as the classical submerged vs. the ALI exposure with regard to the transcriptional response induced by a well-known test substance, the α-quartz Min-U-Sil5. To achieve this goal, we exposed cell cultures to Min-U-Sil5 at the ALI or under submerged conditions to assess and compare transcriptional toxicity profiles. It was ensured that cell lines, cell densities and applied doses were the same under ALI and submerged culture conditions to allow for best possible comparison. 

Based on some recent studies, it was presumed that cells cultivated at an ALI would be more sensitive towards particles [33,34]. Unexpectedly and most strikingly, with respect to mono-cultures, the effect of the quartz particles appears to be far more pronounced after submerged rather than ALI exposure. Especially the extent of activation of inflammatory genes was higher under classical submerged conditions, when comparing the A549 mono-cultures. For example, *IL-8* expression at the highest quartz dose was less than doubled in the ALI mono-culture while being increased 8-fold under submerged conditions after 24 h. Expression of the superoxide dismutase 2 (SOD2) coding gene followed a similar pattern, with expression reaching a maximum of less than 1.5-fold at the ALI and 3-fold under submerged conditions. This higher sensitivity after submerged exposure was partly seen in the co-culture as well, although far less pronounced and with some relevant exceptions, such as *COX2, IL-1A,* and *TNF-A* showing a similar degree of activation and *CCL22* and *IL-6* showing a stronger induction at the ALI. Nevertheless, qualitatively, the cells seemed to regulate a similar pattern of gene expression suggesting that the same toxicity pathways are activated under both ALI and submerged conditions in the co-culture. One explanation why cells are less sensitive at the ALI could consist in the production of surfactant, which may protect the cells from the toxic effects of the particles. Surfactant production in ALI grown A549 cells has been reported previously [9,35]. It seemingly had less of an effect on the co-cultures presented in the present study. 

Generally speaking, the effects of Min-U-Sil5 on the gene expression profiles of the applied cell models were rather modest when compared to highly toxic particles such as copper oxide [36,37,38,39]. This is especially evident considering the high quartz doses, which were able to produce a strong concentration-dependent cytotoxicity in dTHP-1 cells and a moderate cytotoxicity in A549 mono- and A549/dTHP-1 co-cultures under submerged conditions [27]. The gene induced most strongly after 24 h was *IL-8,* with an 8-fold, 32-fold, and 16-fold induction in submerged A549 mono-cultures, dTHP-1 mono-cultures and A549/dTHP-1 co-cultures, respectively. In a similar study employing a 3D model consisting of A549, dTHP-1, and endothelial cells, the induction of inflammatory gene expression (namely the genes *IL-8, IL-6, IL-1A, IL-1B,* and *TNF-A*) rose up to 100 to 500-fold, depending on the gene. However, it has to be noted, that the deposited doses in this particular study were more than three times higher than the applied doses within the present work, with the maximum dose reaching 192 µg/cm^2^. The number of THP-1 cells seeded into the apical compartment in the published study was higher as well, which might contribute to a stronger inflammatory response [10]. Additionally, the lower responses in the present study upon ALI exposure could be due to the formation of a protein corona comprised of BSA, which was intentionally added to stabilize particle suspensions following the NANOGENOTOX protocol [40]. Similarly, FBS proteins from the surrounding media in submerged exposure could mask the reactive silica surface, thus suppressing adverse effects. The alleviating effect of the protein corona on silica toxicity has already been reported in several studies [41,42,43,44,45].

In submerged A549 mono-cultures, other notably induced genes included *IL-6* from the “inflammation” cluster, *NFKB2* and *SOD2* from the “oxidative stress response” cluster and pro-apoptotic DNA damage sensor *DDIT3*. Whereas *IL-6* induction was significant in the submerged cultures at the highest dose, it could not be quantified in ALI cultures at all. Furthermore, an initial two-fold repression of the gene was observed at the lowest dose. This could indicate the activation of anti-inflammatory cytokines, such as IL-4, at lower quartz doses [46]. One pathway of quartz toxicity can be identified as the NF-κB signaling pathway, since, alongside *IL-6,* the induction of another target gene of this pathway was observed: *SOD2*. This can be narrowed down to the non-canonical variant of the pathway, as the upregulation of *NFKB2*, the gene coding for p100 which is processed to the p52 subunit, was also observed. However, upregulation of *NFKB* genes per se does not necessarily result in the whole activation of the pathway because NF-κB subunits need to be post-translationally modified and processed to become fully active and/or translocate to the nucleus [31]. The upregulation of *DDIT3*, a gene coding for the DNA damage-inducible transcript 3, suggested the induction of DNA damage [47], although the corresponding protein is also a part of pro-apoptotic signaling and induced by various stimuli such as hypoxia and ER stress [48]. However, the deviation between experiments was high and no further DNA damage markers were induced. Additionally, no formation of DNA strand breaks was detected in ALI-grown cells via Alkaline Unwinding. This seems to contradict studies that reported DNA damage after exposure to Min-U-Sil5 [23] or NIST quartz [49], although both studies used shorter incubation times, and DNA strand breaks may have been repaired after 24 h. Thus, for A549 cultures the transcriptional response seems to be primarily controlled by the NF-κB pathway. Publications addressing comprehensive gene expression analysis after quartz exposure are scarce. Nevertheless, a similar conclusion was made for rat epithelial cells exposed to DQ12 quartz, although the focus was set on the canonical NF-κB pathway [14]. Moreover, the induction of IL-6 and IL-8 by quartz exposure was observed also on the protein level in other studies using A549 [50,51] and BEAS-2B cells [52].

In dTHP-1 mono-cultures, a qualitatively similar response was observed, with *IL-8* showing the highest induction, and a variety of additional genes being activated. Since the doses applied to the cultures were ten times lower than in the A549 based cultures, the macrophages appeared more susceptible to quartz as a toxicant. Activation of the NF-κB pathway was apparent as well, with *NFKB1* (p50), *NFKB2* (p52), *NFKBIA* (NFKB inhibitor α), and *SOD2* showing an up to four-fold activation. Since both the genes encoding p50 and p52 were up-regulated, both the canonical and non-canonical pathways seemed to be activated. Induction of the inhibitor *NFKBIA* can also be understood as an activation of the pathway, since it is induced by the NF-κB dimer itself through a negative feedback mechanism [53]. The involvement of the NF-κB pathway and oxidative stress in alveolar macrophages after quartz exposure was extensively summarized by Castranova [54], as well as its role in signaling between macrophages and epithelial cells, which becomes relevant for further discussion of results obtained from the co-culture. A broader inflammatory response in the dTHP-1 cells can be observed in comparison to the A549 cultures, as *COX2* (cyclooxygenase 2), *IL-1A* (interleukin-1α), and *IL-1B* (interleukin-1β) were induced more prominently. While all of these genes are downstream targets of the NF-κB pathway [55], they could also be linked to other mechanisms. COX2 and IL-1α can also be regulated by other cytokines such as tumor necrosis factor α (TNF-α) [56,57] or transcription factors such as activator protein 1 (AP-1), which is additionally confirmed by the slight activation of the *JUN* gene in our work [58]. Indeed, in a previous study on the toxicity of nanosilica, activation of c-Jun and induction of TNF-α by the mitogen-activated protein kinase pathway was demonstrated, which precedes the onset of inflammatory gene expression [59]. At the same time, the increased expression of IL-1β in the present study is an indication of NLRP3 inflammasome involvement [60], which was already postulated in another publication [24]. For macrophage-like cells only, it can be summarized that the involvement of several mechanisms is likely. This has been reported previously, with Min-U-Sil5 activating the NF-κB and NLRP3 pathways at the same time [61] and microsilica mediating the transcription of *IL-1B* and *TNF-A* in dTHP-1 cells [62].

For A549/dTHP-1 co-cultures exposed for 24 h, many of the reactions observed in both mono-cultures were amplified, with the modulation of some additional genes, especially in the “inflammation” cluster. As already mentioned, not all induced genes followed the same pattern of a higher activation after submerged exposure to Min-U-Sil5, as it was observed in the A549 mono-cultures. While some genes, such as *IL-1B*, *IL-8, NFKB2,* and *SOD2,* displayed a higher up-regulation after submerged exposure, some showed a quantitatively similar (*COX2, IL1A*) or a higher level of activation at the ALI (*CCL22, IL-6, TNF-A*). However, the general response remained the same for both exposure types on a qualitative level. Similar to both submerged mono-cultures, a clear involvement of the NF-κB pathway was observed, via the activation of *NFKB2*, *NFKBIA*, *SOD2,* and inflammatory genes such as *IL-6*, *IL-8*, *IL-1A/B,* and *COX2*. The latter genes showed a stronger activation when compared to both mono-cultures, which suggests a synergistic interaction between the two different cell types. Presumably, the particles undergo phagocytosis by dTHP-1 cells, which in turn react with the release of several cytokines. The combination of particle exposure and cytokines released by the macrophages has a stronger effect on the A549 cells than the particles alone, which leads to a rise in inflammatory gene expression. Even though A549 and dTHP-1 cells were not separated for gene expression analysis, A549 cells make up the overwhelming majority of the cells because of the difference in seeding numbers (A549: dTHP-1 = 10: 1 at the time of exposure) and the continuous proliferation of the epithelial cells post-incubation. Consequently, most of the mRNA isolated for gene expression analysis derives from the A549 cells. *CCL22*, a gene coding for the macrophage-derived chemokine (MDC) is another notable gene induced only in the co-culture. As the name suggests, it is only produced by macrophages [63]. As such, it was surprisingly induced more prominently in the co-culture than in the dTHP-1 mono-culture. *CCL22* can be induced by lipopolysaccharide, IL-1 and TNF-α [64], the latter of which is also present in the co-culture. For both *CCL22* and *TNF-A,* activation was more pronounced at the ALI, which sets these genes in clear contrast to the other genes investigated. Another cluster that deviated strongly from both mono-cultures was “metal homeostasis”. Especially *MT1X* and *MT2A*, genes coding for metallothioneins, showed a significant two-fold induction at the highest dose after submerged exposure. Since this effect was only observed in the co-culture and Min-U-Sil5 is a quartz with a high purity of 99%, it is unlikely that it is provoked from metal impurities in the material. Nevertheless, metallothioneins act also as scavengers for ROS [65] and can be activated by various stimuli such as oxidative stress and cytokines [66].

Qualitatively similar results with regard to inflammation were reported for 3D models composed of A549/dTHP-1 and endothelial cells [10,11] and in primary rat cells [12], exposed to Min-U-Sil5. In another study applying a 3D culture, no cytokine induction was observed, while a slight induction of oxidative stress was noted. The low responses presumably arise from low administered quartz doses [8]. Other than these studies, no in-vitro studies utilized co-cultures as a tool to study quartz toxicity, especially not at the ALI, which highlights the importance of investigating quartz particle toxicity with multidimensional models.

The time-dependent gene expression analysis in the A549/dTHP-1 co-culture allows for a deeper mechanistic insight. In fact, in the aforementioned study with a 3D-culture different time points have been assessed as well [10]. In the present study, the differences between specific time points were quite pronounced. The biggest changes were observed in the “inflammation” cluster, e.g., for the genes *IL-1A* and *IL-1B* with a 125- and 38-fold activation, respectively, after six hours in comparison to 6- and 2.5-fold after 24 h. The high activation of these two genes at earlier time points suggests a strong involvement of IL-1 dependent pathways. The NF-κB related genes also displayed a strong time-dependency, with peaks at similar, early time points, which points to a possible connection. The clear induction of IL-1β also at earlier time points could be an indicator for NLRP3 inflammasome activation. The connection of quartz toxicity and the inflammasome was already established for A549 and 16HBE14o- [23], as well as for BEAS-2B, THP-1, and NHBE cells [24,25] through direct assessment of NLRP3 and caspase 1 activation by western blot and other protein based assays. It is very likely that this signaling pathway is still relevant in a co-culture model composed of the same cells.

In summary and considering the conclusions from the mono-cultures, it is likely that the mechanisms involved in the more complex co-culture ALI model are a combination of several pathways, among others the NF-κB pathway as well as the NLRP3 inflammasome. Most of the observed changes in gene expression support this, with some notable exceptions. Differences between the submerged and ALI models seem to be quantitative rather than qualitative, with similar genes being activated after exposure of the co-culture, but to different extents. More pronounced differences were observed in the A549 mono-cultures, as ALI cultures showed no biologically relevant changes, while submerged cultures exhibited relevant and significant changes in specific genes. In the future, these findings should be confirmed via other, even more comprehensive multi-omics studies already used to assess toxicity of other aerosols [67,68,69] or specifically targeted approaches, especially protein and activity-based assays. A conditioned media approach, separating the different cell types, or cell sorting procedure subsequent to particle exposure could help to elucidate the specific responses of the single cell types as well. This further research will move forward the field of quartz toxicity research and the use of more complex in vitro models in general.

## 4. Materials and Methods

### 4.1. Materials

All chemicals, cell culture media, and supplements were obtained from Sigma–Aldrich Chemie GmBH (Taufkirchen, Germany) or Carl Roth GmbH (Karlsruhe, Germany), except for fetal bovine serum (FBS), which was obtained from Thermo Fisher Scientific GmBH (Dreieich, Germany). Transwell plates and inserts were acquired from Corning (Amsterdam, Netherlands). Other cell culture materials, such as dishes, flasks, and reaction tubes were bought from Sarstedt (Nuembrecht, Germany). Min-U-Sil5 particles were purchased from U.S.Silica (Katy, TX, USA). Endotoxin content was assessed with the LAL Chromogenic Endotoxin Quantitation Kit (ThermoFisher Scientific, Karlsruhe, Germany). Primers for RT-qPCR were synthesized by Eurofins (Ebersberg, Germany). PCR reagents were purchased from Macherey–Nagel (Dueren, Germany), Applied Biosystems (Foster City, WI, USA), Teknova (Hollister, CA, USA), Fluidigm (San Francisco, CA, USA), Bio-Rad (Munich, Germany) and New England Biolabs (Frankfurt am Main, Germany).

### 4.2. Particle Preparation and Characterization

Particles were dispersed employing a variation of the latest NANOGENOTOX protocol for ALI exposures [40]. Briefly, a defined amount of particles were pre-wetted with 30 µL 97% ethanol before adding 5.970 mL 0.05% bovine serum albumin (BSA) solution. Particle suspensions were sonicated using a Branson Analog Sonifier 450 (Brookfield, WI, USA) for a duration of 13.25 min at 10% amplitude (7197 J). Subsequently, suspensions were aliquoted and frozen at −20 °C. For experiments, suspensions were thawed and sonicated again using a sonication bath for 10 min. For submerged exposure, 10 mg/mL suspensions of Min-U-Sil5 in sterile water were prepared and sonicated for 15 s with a Branson Sonifier 250 (Brookfield, WI, USA). Particle properties were assessed via DLS, using the Zetasizer NanoZS (Malvern Panalytical, Herrenberg, Germany). Furthermore, TEM was performed with a UM910 microscope (Zeiss, Oberkochen, Germany). The results from the DLS measurements as well as the TEM images have already been published in our previous work [27].

### 4.3. Cell Culture

A549 cells (ATCC CCL-185) are human epithelial lung adenocarcinoma cells [70], which were kindly provided by Dr. Roel Schins (Leibniz Research Institute for Environmental Medicine, Duesseldorf, Germany). The cells were cultured at 37 °C in a humidified atmosphere of 5% CO_2_ and grown in RPMI-1640 medium, supplemented with 10% fetal bovine serum (FBS), 100 U/mL penicillin and 100 µg/mL streptomycin. Subcultivation was performed two to three times a week. Only passages 17–55 were used for experiments. 

THP-1 cells (ATCC TIB-202) are human monocytic cells, which can be differentiated into a macrophage-like phenotype (dTHP-1) by incubation with phorbol 12-myristate 13-acetate (PMA) [71,72]. They were kindly provided by Dr. Richard Gminski (Albert-Ludwig-University Freiburg, Department of Environmental Health Sciences and Hygiene, Freiburg, Germany). THP-1 cells were cultured in supplemented RPMI (see above). Prior to experiments the cells were differentiated with 30 ng/mL PMA for five days, followed by an incubation with PMA free medium for three to five days. Only passages 3–22 were used for experiments.

### 4.4. Particle Exposure

Prior to particle application the exposure station (Vitrocell^®^ Cloud) was disinfected with 80% ethanol to create a semisterile environment. Cells were seeded 24 h prior to exposure. 12-well transwell plates were used for ALI exposures while submerged experiments were performed in 12- or 24-well plates. The submerged culture has already been described and applied in a previous work assessing the toxicity of CuO particles [36]. A549 cells were seeded at a density of 1.96 × 10^5^ cells/cm^2^ to achieve a tight monolayer at the time of exposure. The cells were given four to six hours to adhere. Afterwards, dTHP-1 cells were seeded on top at a density of 2.95 × 10^4^ cells/cm^2^ for mono-cultures and co-cultures with A549 cells. These densities were determined to ensure a ratio of 10:1 between epithelial cells and dTHP-1 at the moment of exposure, which corresponds to the physiological ratio between epithelial cells and macrophages in a healthy lung [73].

The procedure for ALI exposure with the Cloud was described in detail in [37]. One hour preceding the exposure, the medium was removed from the apical compartment to get the cells accustomed to ALI conditions. Particle suspensions were thawed and re-sonicated, as described above. The transwell inserts with the cell cultures were transferred into the pre-warmed device and exposed to the particle aerosol for 10 min, before transferring them into plates with fresh cell culture medium for post-incubation. The post-incubation period was 24 h, unless stated otherwise. Particle deposition was monitored for 30 more minutes with a quartz crystal microbalance (QCM). The final deposition was determined by calculation of the means of the last 100 data points. Submerged exposures were performed by diluting the particle suspensions to the desired concentrations with cell culture medium after re-sonication. 

After a post-incubation time of 24 h (1, 3, 6, or 24 h for the time-dependent studies) the cells were detached using trypsin solution or accutase and washed with PBS. The resulting pellets were stored at −80 °C until further use.

### 4.5. Gene Expression Analysis via High-Thoughout RT-qPCR

Gene expression analysis was performed as described in [28]. The original gene set was adapted; seven genes coding for inflammatory (*CCL22, COX2, IL-1A, IL-1B, IL-6, IL-8, TNFA*) and fibrotic proteins (*ACTA2, COL1A1, CTNNB1, FN1, OPN, PDGFA, TGFB, TIMP1, VIM*) were added newly and validated. *ACTB, B2M, GAPDH, GUSB,* and *HPRT1* were used as housekeeping genes. All genes and corresponding proteins are depicted in Supplementary Table S3. Briefly, mRNA was isolated from cell pellets using the NucleoSpin RNA Plus Kit (Macherey–Nagel) and quantified with a plate reader. The mRNAs were transcribed into cDNA employing the qScript cDNA Synthesis Kit (QuantaBio). The resulting cDNA was pre-amplified with the help of a pooled primer mix and TaqMan Preamp Master Mix (Applied Biosystems), followed by exonuclease I treatment (New England Biolabs). The PCR was performed in a 96 × 96 Dynamic Array integrated fluidic circuit (Fluidigm). PCR data were analyzed with the Fluidigm Real-Time PCR Analysis and GenEx softwares. Differences in the resulting Cq-values were assessed with the ΔΔCq-method, and displayed as fold-change compared to an untreated control. All gene expression analyses were performed in two technical replicates.

### 4.6. Evaluation of DNA Strand Breaks by Alkaline Unwinding

The alkaline unwinding method was described in detail in previous studies [32,37]. Briefly, cell harvesting and counting were performed as described above, followed by centrifugation of the cells (1300 rpm, 4 min, 4 °C). The resulting pellet was resuspended in cold PBS to reach a concentration of 10^5^ cells/10 µL. The following steps were performed in duplicates. 10 µL of cell suspension and 750 µL of alkaline solution (0.03 M NaOH, 0.02 M Na_2_HPO_4_, 0.9 M NaCl) were added to a glass tube and incubated for 30 min. After the incubation period, 0.1 N HCl was added to reach a pH of 6.8, followed by sonication for 15 s and the addition of 15 µL sodium dodecylsulfate (10%). The samples were frozen for later steps. The samples then underwent a hydroxyapatite chromatography at 60 °C. Differently concentrated potassium phosphate buffers were added to separate single stranded from double-stranded DNA. Finally, the separated DNA was stained with Hoechst 33,258 and quantified via fluorescence measurement (exc.: 360 nm, em.: 455 nm). The amount of double-stranded DNA was calculated as described previously [32].

### 4.7. Statistical Analysis

Cell culture experiments were carried out in duplicates in at least three individual experiments, using three different cell passages. Gene expression experiments were performed in two technical replicates from at least three individual experiments. Data are displayed as means ± SD. Differences between untreated/PBS treated and particle treated samples were assessed by one-way ANOVA followed by Dunnett’s post hoc test. *p*-values below 0.05 (0.01) were considered statistically significant. The data analysis was generated using the Real Statistics Resource Pack software (Version 7.3.2) Copyright (2013–2021) Charles Zaiontz (www.real-statistics.com) Differences between cell lines were assessed by student’s *t*-test. *p*-values below 0.05 were considered statistically significant.

## 5. Conclusions

The aim of this investigation was to assess the mechanisms of quartz toxicity in lung cell culture models differing in complexity and exposure method. For this purpose, air-liquid interface models composed of A549 alveolar epithelial cells in mono-culture and co-culture with macrophage-like dTHP-1 cells were established, exposed to different doses of α-quartz Min-U-Sil5 and finally examined with regard to gene expression of selected targets representing distinct toxicity pathways. The models were compared to their submerged counterparts and a submerged dTHP-1 mono-culture. Generally, there were few differences between submerged and ALI exposure qualitatively, but the magnitude of the toxicological reactions differed significantly, which became especially evident in the A549 mono-cultures. While similar genes were observed to be up-regulated for both exposure types, biologically relevant transcriptional changes only occurred after submerged exposure. The introduction of dTHP-1 cells as part of a co-culture showed a more pronounced response as well, which emphasizes the importance of considering interactions between different cell types in toxicological testing. For both mono- and co-culture models, the NF-κB pathway and NLRP3 inflammasome activation were identified as plausible mediators of quartz toxicity in line with previous findings, and need be studied further in more detail. Time resolved analysis of gene expression profiles in the co-cultures revealed the importance of early signaling events particularly relevant for the induction of inflammatory genes. Therefore, future studies should also focus on the kinetics of transcription profiles to fully capture the different modes of action relevant for particle toxicology. 

This study provides the first comprehensive comparison of transcriptional toxicity profiles of α-quartz Min-U-Sil5 after ALI and submerged exposure along with mono- and co-cultures at the same time. These data highlight differences in the magnitude of results depending on the respective in vitro model, and highlight experimental aspects to be considered in future testing in particle toxicology.

## Data Availability

The data presented in this study are available on request from the first (A.F. and S.F.D.) and corresponding author (A.H. and C.W.) for researchers of academic institutes who meet the criteria for access to the confidential data.

## Supplementary Materials

The following supporting information can be downloaded at: https://www.mdpi.com/article/10.3390/ijms23147773/s1.

## Abbreviations

ALI	Air-liquid interface
AU	Alkaline unwinding
BSA	Bovine serum albumin
*CCL22*	Gene coding for C-C motif chemokine 22
*COX2*	Gene coding for cyclooxygenase 2
*DDIT3*	Gene coding for DNA damage inducible protein
DLS	Dynamic light scattering
dTHP-1	differentiated THP-1 cells
FBS	Fetal bovine serum
HT RT qPCR	High-throughput RT qPCR
*IL-1A/1B/6/8*	Genes coding for interleukin-1α/1β/6/8
*JUN*	Gene coding for jun proto-oncogene
*MT1X/2A*	Genes coding for metallotionein 1X/2A
NF-κB	Nuclear factor κB
NLRP3	NOD-, LRR- and pyrin domain containing protein 3
PMA	Phorbol 12-myristate 13-acetate
QCM	Quartz crystal microbalance
ROS	Reactive oxygen species
*SOD2*	Gene coding for superoxide dismutase 2
TEM	transmission electron microscopy
*TNF-A*	Gene coding for tumor necrosis factor α
*TNFRSF10B*	Gene coding for tumor necrosis factor receptor superfamily, member 10b/death receptor 5
*VEGFA*	Gene coding for vasular endothelial growth factor A
