# Supplementary material for: Gene Expression Profiling of Mono- and Co-Culture Models of the Respiratory Tract Exposed to Crystalline Quartz under Submerged and Air-Liquid Interface Conditions
Source: Int J Mol Sci. 2022 Jul 14;23(14):7773. doi: 10.3390/ijms23147773 (PMC9324045; doi:10.3390/ijms23147773)
Supplement: Supplementary file 1 [file ijms-23-07773-s001.zip › ijms-1800477-supplementary.pdf]

Supplementary information to:

Gene expression profiling of mono- and co-culture models of the respiratory tract exposed to crystalline quartz under submerged and air-liquid interface conditions

Alexandra Friesen[1,†], Susanne Fritsch-Decker[2,†], Matthias Hufnagel[1], Sonja Mülhopt[3], Dieter Stapf[3], Carsten Weiss[2,*] and Andrea Hartwig[1,*]

[1] Department of Food Chemistry and Toxicology, Institute of Applied Biosciences (IAB), Karlsruhe Institute of Technology (KIT), 76131 Karlsruhe, Germany; alexandra.friesen@kit.edu (A.F.); matthias.hufnagel@gmail.com (M.H.)

[2] Institute of Biological and Chemical Systems, Biological Information Processing, Karlsruhe Institute of Technology (KIT), 76344 Eggenstein-Leopoldshafen, Germany; susanne.fritsch-decker@kit.edu

[3] Institute of Biological and Chemical Systems, Biological Information Processing, Karlsruhe Institute of Technology (KIT), 76344 Eggenstein-Leopoldshafen, Germany; sonja.muelhopt@kit.edu (S.M.); dieter.stapf@kit.edu (D.S.)

* Correspondence: andrea.hartwig@kit.edu (A.H.); carsten.weiss@kit.edu (C.W.)

† These authors contributed equally to this work.

Supplementary Table S1: log2-fold changes of all genes in the applied gene set after exposure of A549 and dTHP-1 mono-cultures to Min-U-Sil5 and 24 hours post-incubation. A549 ALI cultures were exposed to 15.8 ± 0.8 (low), 29.1 ± 2.4 (mid), and 59.4 ± 2.0 µg/cm² (high), A549 submerged cultures to 15 (low), 30 (mid) and 60 µg/cm² (high) and dTHP-1 submerged cultures to 1.5 (low), 3 (mid), and 6 µg/cm² (high) Min-U-Sil5. The means of at least three independent experiments are displayed.

	log2-fold change								
	**A549 ALI (dose)**			**A549 submerged (dose)**			**dTHP-1 (dose)**		
	low	mid	high	low	mid	high	low	mid	high
AHR	-0,12	0,20	0,13	-0,07	-0,14	-0,20	0,16	0,01	0,08
CYP1A1	-0,33	0,14	-0,51	0,17	0,55	0,30	1,09	1,31	2,03
NQO1	0,02	-0,05	0,09	-0,02	-0,02	0,09	0,17	0,38	0,62
CCL22	0,81	0,01	-0,48	0,26	-0,20	1,24	0,07	0,09	0,26
COX2	-0,13	0,04	0,28	0,17	0,26	0,41	0,15	0,15	0,64
IL-1A	-0,31	0,17	0,60	-0,15	-0,50	0,35	0,10	0,34	0,76
IL-1B	0,04	-0,62	-0,86	0,23	0,47	0,52	0,15	0,46	0,97
IL-6	-	-	0,13	-1,00	0,30	1,46	-0,29	0,74	0,28
IL-8	0,13	0,26	0,45	0,29	1,18	2,46	1,69	3,00	4,58
TNF-A	-	-	-	-	-	-	0,04	0,10	0,35
ACTA2	-0,11	-0,05	-0,06	0,05	-0,11	-0,35	-0,34	-0,13	0,08
COL1A1	-0,58	-0,12	-1,48	-0,22	-0,02	0,03	0,06	0,21	0,08
CTNNB1	-0,16	-0,11	0,24	0,17	0,10	0,06	0,16	0,09	0,13
FN1	0,03	0,20	0,17	-0,16	-0,41	-0,89	-0,14	-0,13	-0,40
OPN	0,12	-0,13	0,13	0,17	0,20	0,43	-0,06	-0,08	-0,24
PDGFA	-0,15	0,02	0,19	0,02	0,05	0,12	0,03	0,00	-0,20
TGFB	-	-	-0,28	-0,08	0,08	0,09	0,10	-0,07	-0,20
TIMP1	-0,04	0,20	0,15	-0,01	-0,03	0,02	-0,21	-0,08	-0,14
VIM	0,03	0,29	0,04	-0,19	-0,13	-0,15	-0,11	-0,38	-0,33
FTH1	-0,19	0,62	0,21	0,53	0,08	-1,02	-0,10	-0,67	0,09
MT1X	0,01	0,06	0,01	-0,06	0,28	0,51	-0,07	-0,08	0,14
MT2A	-0,03	0,28	0,02	0,22	0,28	0,40	-0,08	-0,18	0,37
SLC30A1	-0,11	0,00	0,08	0,07	0,00	-0,02	0,04	-0,19	-0,30
TFRC	-0,13	0,04	0,25	0,14	0,09	0,19	0,19	0,24	0,48
CAT	0,06	0,04	-0,02	-0,13	-0,12	-0,13	0,03	0,01	-0,15
G6PD	-0,12	-0,10	0,12	0,11	-0,01	-0,15	0,08	0,09	0,15
GCLC	0,03	0,07	0,15	-0,02	-0,19	-0,25	0,09	-0,02	-0,22
GPX1	0,07	0,07	-0,10	0,06	0,18	0,13	0,03	0,10	0,19
GPX2	-0,01	0,06	0,09	-0,19	-0,36	-0,43	-	-	-
GSR	-0,01	-0,12	0,14	0,12	0,08	0,14	0,21	0,36	0,44
HMOX1	0,02	0,07	0,30	0,18	0,21	0,23	-0,02	0,19	0,28
HSPA1A	0,09	0,20	0,26	0,08	0,22	0,17	0,05	0,05	0,20
KEAP1	-0,18	-0,11	-0,03	0,03	-0,10	-0,24	0,12	0,19	0,04
MAP3K5	-0,17	0,12	0,09	0,04	-0,06	-0,03	0,02	-0,10	-0,26
NFE2L2	0,03	0,20	0,18	-0,02	-0,03	-0,02	0,19	0,16	0,25
NFKB1	-0,09	-0,06	0,05	0,03	0,18	0,30	0,32	0,54	0,85
NFKB2	-0,28	-0,07	0,11	0,48	0,80	1,18	0,40	0,79	1,25
NFKBIA	-0,21	0,24	0,09	0,30	0,44	-0,04	0,31	0,25	0,78
PRDX1	0,03	0,22	0,04	-0,04	0,03	0,19	-0,01	0,22	0,46
SOD1	0,27	-0,40	0,09	-0,01	-0,07	0,03	-0,12	-0,11	-0,11

SOD2	0,11	0,15	0,40	0,37	0,68	1,29	0,50	0,96	1,92
TXN	0,19	0,27	-0,01	-0,04	0,16	0,31	0,08	0,02	0,28
TXNRD1	-0,02	0,02	0,22	0,07	0,01	0,09	0,32	0,56	0,81
APAF1	-0,25	-0,08	0,11	0,00	0,08	-0,21	0,11	0,05	-0,18
BAX	0,00	-0,10	0,09	0,13	0,17	0,18	0,02	0,25	0,33
BBC3	-0,02	-0,39	-0,57	0,47	0,02	0,05	0,14	0,16	0,15
BCL2	-0,11	-0,08	0,10	0,24	0,28	0,19	0,28	0,36	0,38
BTRC	-0,06	0,02	0,07	0,05	-0,10	-0,21	0,07	0,03	0,00
CCND1	-0,03	-0,09	0,23	-0,06	-0,07	0,07	0,19	0,25	0,51
CDKN1A	-0,13	0,00	0,17	0,13	0,18	0,37	0,11	0,20	0,12
CDKN1B	-0,05	-0,01	0,15	0,03	0,02	-0,07	0,03	-0,04	-0,23
CDKN2B	-	-	-	-	-	-	-	-	-
E2F1	-0,05	-0,02	0,16	0,00	0,11	0,17	0,15	0,23	0,40
EGFR	-0,11	-0,12	0,08	0,17	0,28	0,24	-0,12	0,09	0,94
JUN	-0,23	-0,07	0,28	0,10	0,32	0,40	0,33	0,61	0,86
MDM2	-0,05	-0,02	0,06	-0,03	-0,06	-0,08	0,14	0,11	0,15
MYC	-0,06	0,12	0,30	0,14	0,01	-0,06	0,21	0,06	0,11
PLK3	0,06	0,14	0,17	0,07	0,16	-0,14	0,15	0,31	0,54
PMAIP1	-0,07	0,15	0,16	0,02	0,11	0,27	0,11	0,20	0,38
PPM1D	-0,08	-0,07	-0,02	-0,08	0,07	0,02	0,13	0,07	-0,03
SIRT2	0,02	0,04	0,14	0,28	0,45	0,41	0,01	0,19	0,02
TNFRSF10B	-0,06	-0,04	0,21	0,10	0,27	0,31	0,18	0,43	0,43
VEGFA	-0,01	0,33	0,36	0,33	0,32	-0,48	0,61	0,55	1,40
XIAP	-0,18	-0,05	0,11	0,06	-0,08	-0,16	0,18	0,18	0,16
APEX1	0,00	0,13	-0,04	-0,09	-0,17	-0,22	-0,06	0,02	-0,12
ATM	-0,05	-0,06	0,03	-0,10	-0,14	-0,26	0,05	-0,06	-0,38
ATR	-0,04	0,24	0,00	-0,12	-0,05	-0,07	-0,11	-0,10	-0,03
BRCA1	-0,07	0,11	0,04	-0,18	-0,05	-0,07	0,13	0,06	0,17
BRCA2	-0,12	0,12	0,00	-0,01	0,09	0,06	0,16	0,13	0,28
DDB1	-0,13	-0,07	0,05	0,03	0,01	0,03	0,18	0,43	0,41
DDB2	0,05	-0,02	-0,17	-0,22	-0,12	-0,13	0,14	0,32	0,33
DDIT3	0,42	0,78	1,07	0,76	0,87	0,21	0,28	0,25	1,00
ERCC1	0,08	0,13	0,02	-0,15	-0,01	0,06	-0,05	-0,10	-0,24
ERCC4/XPF	-0,02	-0,03	0,11	-0,03	0,02	0,02	0,10	0,06	-0,01
ERCC5/XPG	-0,07	-0,03	-0,04	-0,11	-0,16	-0,32	0,11	0,08	-0,10
GADD45A	-0,03	0,08	0,00	-0,10	0,22	0,39	0,42	0,37	0,48
LIG1	-0,14	0,05	-0,07	0,02	0,02	-0,09	0,03	0,18	0,34
LIG3	-0,10	-0,10	0,07	0,02	-0,05	-0,18	0,16	0,26	-0,09
MGMT	-0,01	0,26	-0,02	0,01	0,07	-0,04	-0,02	0,04	0,01
MLH1	-0,06	-0,01	0,01	-0,05	-0,10	-0,10	0,14	0,24	0,22
MSH2	0,03	0,25	0,03	-0,15	-0,17	-0,14	-0,03	0,03	0,15
PARP1	-0,07	-0,01	0,05	-0,08	-0,10	-0,22	0,16	0,19	0,08
PCNA	0,12	0,25	0,07	-0,11	0,02	0,16	0,14	0,19	0,33
POLB	0,02	0,10	-0,27	0,09	0,19	-0,16	0,15	0,05	0,00
POLQ	-0,24	-0,12	-0,07	0,00	0,22	0,31	-0,02	0,00	-0,06
RAD50	-0,04	0,07	0,07	0,00	0,01	0,02	0,06	0,13	0,00
RAD51	0,06	0,17	0,04	-0,08	-0,01	0,05	0,12	0,29	0,43
RRM2B	-0,16	-0,01	0,14	0,02	-0,02	-0,04	0,08	-0,01	-0,24
XPA	0,11	0,14	-0,01	-0,17	-0,13	-0,17	0,04	-0,04	-0,09
XPC	-0,14	-0,04	0,00	0,03	-0,11	-0,19	0,09	0,12	-0,09

Supplementary Table S2: log2-fold changes of all genes in the applied gene set after exposure of A549/dTHP-1 co-cultures to Min-U-Sil5 and 24 hours or 1, 3, and 6 hours post-incubation. ALI co-cultures were exposed to 15.9 ± 0.6 (low), 32.2 ± 2.9 (mid), and 55.9 ± 2.9 µg/cm² (high), submerged co-cultures to 15 (low), 30 (mid) and 60 µg/cm² (high) and time-dependent co-cultures to 56.9 ± 2.94 µg/cm² (high) Min-U-Sil5. The means of at least three independent experiments are displayed.

	log2-fold change								
	**A549/dTHP-1 ALI (dose)**			**A549/dTHP-1 submerged (dose)**			**A549/dTHP-1 ALI (time)**		
	low	mid	high	low	mid	high	1h	3h	6h
AHR	-0,04	-0,04	-0,15	-0,17	-0,13	-0,03	-0,23	0,04	0,08
CYP1A1	-1,18	-0,10	-0,04	0,06	0,34	0,74	0,13	-0,19	-0,48
NQO1	-0,12	-0,14	-0,31	0,05	-0,11	-0,39	0,03	-0,12	-0,18
CCL22	1,14	1,18	1,65	0,39	0,66	-0,16	-0,88	0,46	0,88
COX2	0,18	0,42	1,26	0,54	1,27	1,51	-0,58	1,47	1,94
IL-1A	1,82	0,23	2,50	0,79	1,49	2,43	-0,43	4,97	6,96
IL-1B	1,15	0,70	1,11	0,99	1,32	2,64	1,00	3,69	5,23
IL-6	0,82	1,49	3,21	2,24	2,26	2,47	-0,31	3,61	4,58
IL-8	0,44	1,09	2,75	1,82	1,78	3,92	-0,35	2,65	3,33
TNF-A	0,38	0,66	1,26	0,68	-0,18	0,47	1,80	3,02	2,62
ACTA2	-0,09	-0,37	-0,26	0,21	-0,12	-0,25	-0,04	-0,07	-0,17
COL1A1	-0,34	0,24	0,10	0,21	-0,22	0,92	-0,31	0,11	-0,95
CTNNB1	-0,01	-0,24	0,17	0,22	0,22	0,48	-0,01	-0,24	0,22
FN1	0,02	-0,32	-0,36	-0,08	-0,08	-0,14	-0,56	-0,07	0,09
OPN	0,26	0,18	-0,16	-0,18	0,16	0,10	-0,70	0,44	0,17
PDGFA	0,02	-0,05	-0,09	0,36	0,14	0,42	0,08	-0,48	-0,56
TGFB	0,13	-0,02	0,30	0,20	0,18	0,20	-0,11	-0,04	0,19
TIMP1	0,05	0,17	0,17	0,02	-0,06	0,04	0,14	0,51	0,10
VIM	-0,08	0,05	-0,20	-0,08	-0,24	-0,31	0,25	0,31	0,00
FTH1	0,23	0,42	0,47	0,21	0,11	1,01	0,73	-1,14	-0,35
MT1X	-0,05	0,10	0,42	0,43	0,78	1,10	-0,39	0,47	0,23
MT2A	0,06	0,31	0,53	0,19	0,23	1,11	0,07	0,19	0,19
SLC30A1	0,06	-0,03	0,14	0,17	0,10	0,22	-0,19	-0,45	-0,14
TFRC	0,15	-0,08	-0,03	0,15	0,15	0,58	-0,19	-0,56	0,16
CAT	0,00	-0,10	-0,14	0,01	-0,16	-0,20	-0,03	0,00	-0,25
G6PD	0,02	-0,20	-0,10	0,06	-0,26	-0,26	0,17	-0,22	-0,20
GCLC	-0,05	-0,22	-0,23	-0,07	-0,31	-0,22	-0,07	-0,12	0,06
GPX1	0,06	0,01	-0,21	-0,07	-0,05	-0,24	0,04	-0,08	-0,03
GPX2	-0,03	0,04	-0,25	-0,31	-0,22	-0,84	-0,15	0,37	-0,07
GSR	-0,05	-0,15	0,02	0,06	0,00	0,18	-0,11	-0,38	-0,22
HMOX1	0,05	-0,05	-0,06	0,06	-0,05	0,03	0,21	-0,42	-0,41
HSPA1A	-0,09	0,10	0,09	0,11	0,01	0,07	0,07	-0,04	0,02
KEAP1	-0,04	-0,24	-0,06	0,11	-0,21	-0,28	0,28	-0,64	-0,12
MAP3K5	0,20	0,18	0,20	0,02	-0,04	0,26	0,05	-0,03	-0,11
NFE2L2	0,03	0,07	0,10	0,13	0,12	0,36	-0,20	0,38	0,21
NFKB1	-0,01	-0,06	0,28	0,31	0,26	0,67	-0,07	-0,02	1,15
NFKB2	0,03	0,15	0,66	0,87	0,72	1,51	0,11	-0,32	1,09
NFKBIA	-0,06	0,01	0,88	0,76	0,67	1,57	0,34	1,75	0,92
PRDX1	0,00	0,18	0,25	-0,03	-0,02	0,01	0,13	0,42	-0,17
SOD1	-0,79	0,01	-0,08	0,08	-0,87	0,37	0,10	0,34	-0,03
SOD2	0,64	1,32	2,28	1,42	1,88	3,29	-0,42	0,98	2,07

TXN	0,10	0,29	0,46	0,18	0,29	0,30	0,06	0,57	-0,09
TXNRD1	0,03	0,02	0,28	-0,04	-0,12	0,39	-0,16	0,12	0,19
APAF1	-0,05	-0,36	-0,05	0,15	0,03	0,22	0,03	-0,43	0,05
BAX	-0,18	-0,05	-0,13	0,10	-0,04	-0,10	0,12	-0,08	-0,08
BBC3	-0,11	-0,67	-0,81	0,22	-0,25	-0,19	-0,20	-0,11	1,20
BCL2	-0,07	-0,32	-0,51	-0,06	-0,19	-0,40	-0,06	-0,56	-0,27
BTRC	0,04	-0,16	-0,02	0,10	-0,18	-0,01	0,13	-0,23	-0,15
CCND1	-0,05	-0,16	0,04	0,04	-0,16	0,02	-0,07	-0,13	0,07
CDKN1A	-0,01	-0,15	0,03	0,26	0,17	0,27	0,00	0,10	0,57
CDKN1B	0,06	-0,03	0,03	0,20	0,13	0,24	0,04	-0,44	-0,10
CDKN2B	-	-	-	-	-	-	-	-	-
E2F1	-0,01	-0,25	0,31	-0,13	-0,22	-0,14	0,09	-0,19	-0,52
EGFR	-0,05	-0,25	0,30	0,34	0,41	0,86	-0,10	-0,37	0,41
JUN	-0,02	-0,25	0,34	0,51	0,03	0,49	0,38	0,05	0,25
MDM2	-0,09	-0,11	0,21	0,23	0,13	0,28	0,05	0,02	0,02
MYC	0,01	-0,02	0,36	0,20	-0,01	0,14	-0,18	-0,37	0,09
PLK3	0,25	0,15	0,32	0,43	0,27	0,31	0,04	0,32	0,49
PMAIP1	0,01	0,20	0,21	0,02	0,05	0,17	-0,45	0,26	0,19
PPM1D	-0,14	-0,24	-0,05	0,07	0,19	0,02	-0,01	-0,16	-0,20
SIRT2	0,07	-0,09	-0,02	0,09	-0,06	0,04	0,09	-0,12	-0,11
TNFRSF10B	0,05	-0,07	0,47	0,24	0,24	0,81	-0,29	0,05	0,30
VEGFA	0,07	0,01	0,49	0,46	0,19	1,09	0,17	-0,13	0,19
XIAP	-0,05	-0,08	-0,06	0,02	-0,19	0,05	0,01	-0,25	0,17
APEX1	0,02	0,15	0,00	0,01	-0,21	-0,19	0,16	0,13	-0,12
ATM	-0,08	-0,20	-0,22	0,08	-0,19	-0,05	-0,08	-0,16	-0,14
ATR	-0,04	0,13	0,11	0,07	-0,11	-0,21	0,10	0,14	-0,19
BRCA1	-0,08	-0,12	-0,09	-0,07	-0,07	-0,07	0,12	-0,09	-0,29
BRCA2	-0,02	0,00	0,01	-0,07	-0,08	-0,08	-0,01	-0,22	-0,45
DDB1	-0,05	-0,26	0,06	0,06	-0,03	0,06	0,04	-0,20	-0,12
DDB2	-0,08	-0,27	-0,24	-0,27	-0,36	-0,60	-0,14	-0,07	-0,14
DDIT3	-0,14	0,66	0,99	0,39	0,33	0,80	0,11	-0,53	-0,42
ERCC1	-0,05	0,12	0,09	-0,02	0,00	0,01	-0,01	0,41	0,09
ERCC4/XPF	-0,10	0,03	0,24	0,02	0,02	0,15	-0,05	-0,02	-0,09
ERCC5/XPG	-0,15	-0,21	-0,10	0,04	-0,18	-0,16	-0,09	0,06	-0,26
GADD45A	-0,05	-0,02	-0,51	0,04	0,14	-0,32	-0,19	0,24	0,67
LIG1	-0,03	-0,22	-0,18	0,04	-0,26	-0,26	0,24	-0,53	-0,45
LIG3	-0,05	-0,31	-0,26	0,10	-0,09	-0,11	0,12	-0,35	-0,03
MGMT	-0,03	0,07	-0,07	-0,06	-0,17	-0,38	0,18	0,37	0,00
MLH1	-0,02	-0,09	0,09	0,00	-0,24	-0,15	0,08	-0,09	-0,30
MSH2	0,01	0,11	-0,06	0,01	-0,16	-0,17	0,17	0,05	-0,25
PARP1	-0,03	-0,13	-0,17	0,01	-0,25	-0,16	0,01	-0,32	0,00
PCNA	0,12	0,11	0,35	0,02	0,11	-0,08	-0,28	0,23	-0,06
POLB	0,07	-0,29	0,00	0,14	-0,22	-0,07	-0,05	-0,20	-0,16
POLQ	-0,09	-0,26	0,11	0,16	0,13	-0,02	0,27	-0,79	-0,39
RAD50	-0,08	0,02	-0,26	0,02	-0,01	0,12	0,08	-0,10	-0,08
RAD51	0,08	0,12	0,11	-0,07	-0,13	-0,20	0,15	-0,09	-0,16
RRM2B	-0,13	-0,25	-0,14	0,07	-0,04	0,13	-0,04	-0,34	-0,11
XPA	-0,07	0,08	-0,52	-0,01	-0,08	-0,08	-0,08	0,23	0,04
XPC	-0,02	-0,19	-0,07	0,04	-0,23	-0,11	0,11	-0,23	-0,23

Supplementary Table S3: Complete list of genes utilized in the HT RT-qPCR with their corresponding proteins.

Cluster	Gene	Corresponding protein
Xenobiotic metabolism	*AHR*	aryl hydrocarbon receptor
	CYP1A1	cytochrome P450, family 1, subfamily A, polypeptide 1 (CYP1A1)
	NQO1	NAD(P)H dehydrogenase, quinone 1 (NQO1)
Inflammation	*CCL22*	C-C motif chemokine 22 (CCL22)
	COX2	prostaglandin-endoperoxide synthase 2 (PTGS2)/ cyclooxygenase-2 (COX-2)
	IL-1A	interleukin-1α (IL-1α)
	IL-1B	interleukin-1β (IL-1β)
	IL-6	interleukin-6 (IL-6)
	IL-8	interleukin-8 (IL-8)
	TNF-A	tumor necrosis factor α (TNF-α)
Fibrosis	*ACTA2*	alpha smooth muscle actin (α-SMA)
	COL1A1	collagen, type I, alpha 1 (COL1A1)
	CTNNB1	β-catenin (CTNNB)
	FN1	fibronectin1 (FN1)
	OPN	osteopontin (OPN)
	PDGFA	platelet-derived growth factor subunit A (PDGF-A)
	TGFB	transforming growth factor β (TGF-β)
	TIMP1	tissue inhibitor of metalloproteinase (TIMP1)
	VIM	vimentin (VIM)
Metal homeostasis	*FTH1*	ferritin, heavy polypeptide 1 (FTH1)
	MT1X	metallothionein 1X (MT1X)
	MT2A	metallothionein 2A (MT2A)
	SLC30A1	solute carrier family 30 (zinc transporter), member 1 (ZnT1)
	TFRC	transferrin receptor (TFR)
Oxidative stress response	*CAT*	catalase (CAT)
	G6PD	glucose-6-phosphate dehydrogenase (G6PD)
	GCLC	glutamate-cysteine ligase, catalytic subunit (GCL)
	GPX1	glutathione peroxidase 1 (GPX1)
	GPX2	glutathione peroxidase 2 (GPX2)
	GSR	glutathione reductase (GSR)
	HMOX1	heme oxygenase (decycling) 1 (HO1)
	HSPA1A	heat shock 70kDa protein 1A (hsp70)
	KEAP1	kelch-like ECH-associated protein 1 (Keap1)
	MAP3K5	mitogen-activated protein kinase kinase kinase 5 (MAP3K5/ASK1)
	NFE2L2	nuclear factor, erythroid 2-like 2 (Nrf2)
	NFKB1	nuclear factor of kappa light polypeptide gene enhancer in B-cells 1 (p50/p105)

	NFKB2	nuclear factor of kappa light polypeptide gene enhancer in B-cells 2 (p49/p100)
	NFKBIA	nuclear factor of kappa light polypeptide gene enhancer in B-cells inhibitor,alpha (IKBA)
	PRDX1	peroxiredoxin 1 (Prx1)
	SOD1	superoxide dismutase 1, soluble (SOD1)
	SOD2	superoxide dismutase 2, mitochondrial (SOD2/MnSOD)
	TXN	thioredoxin (Txr)
	TXNRD1	thioredoxin reductase 1 (TxrR)
Apoptosis and cell cycle regulation	*APAF1*	apoptotic protease activating factor (APAF1)
	BAX	bcl2-associated x protein (bax)
	BBC3	p53 up-regulated modulator of apoptosis (PUMA)
	BCL2	b-cell cll/lymphoma 2 (bcl-2)
	BTRC	transducin repeat containing E3 ubiquitin protein ligase, beta (β-TrCP)
	CCND1	cyclin D1 (CCND1)
	CDKN1A	cyclin-dependent kinase inhibitor 1A (p21)
	CDKN1B	cyclin-dependent kinase inhibitor 1B (p27)
	CDKN2B	cyclin-dependent kinase inhibitor 2B (p15)
	E2F1	E2F transcription factor 1 (E2F1)
	EGFR	epidermal growth factor receptor (EGFR)
	JUN	jun proto-oncogene (c-jun)
	MDM2	Mouse double minute 2 hom. proto-onco-gene, E3 ubiquitin protein ligase (Mdm2)
	MYC	v-myc avian myelocytomatosis viral oncogene homolog (c-myc)
	PLK3	polo-like kinase 3 (PLK3)
	PMAIP1	phorbol-12-myristate-13-acetate-induced protein 1 (Noxa)
	PPM1D	protein phosphatase, mg2+/Mn2+ dependent, 1D (PPM1D)
	SIRT2	sirtuin 2 (SIRT2)
	TNFRSF10B	tumor necrosis factor receptor superfamily, member 10b (DR5)
	VEGFA	vascular endothelial growth factor A (VEGFA)
	XIAP	X-linked inhibitor of apoptosis (XIAP)
DNA damage response and repair	*APEX1*	apurinic-apyrimidinic endonuclease 1 (APEX1/Ref-1)
	ATM	ataxia telangiectasia mutated (ATM)
	ATR	ataxia telangiectasia and Rad3-related protein (ATR)
	BRCA1	breast cancer 1, early onset (BRCA1)
	BRCA2	breast cancer 2, early onset (BRCA2)
	DDB1	damage-specific DNA binding protein 1 (DDB1)
	DDB2	damage-specific DNA binding protein 2 (DDB2)
	DDIT3	growth arrest and DNA damage-inducible protein (GADD153)

ERCC1	excision repair cross-complementation group 1 (ERCC1)
ERCC4/XPF	excision repair cross-complementation group 4 (XPF)
ERCC5/XPG	excision repair cross-complementation group 5 (XPG)
GADD45A	growth arrest and DNA-damage-inducible, alpha (GADD45A)
LIG1	ligase I, DNA, ATP-dependent (LIG1)
LIG3	ligase III, DNA, ATP-dependent (LIG3)
MGMT	O-6-methylguanine-DNA methyltransferase (MGMT)
MLH1	mutL homolog 1 (MLH1)
MSH2	mutS homolog 2 (MSH2)
PARP1	Poly (ADP-ribose) polymerase 1 (PARP1)
PCNA	proliferating cell nuclear antigen (PCNA)
POLB	polymerase (DNA directed), beta (POLB)
POLQ	
RAD50	RAD50 homolog (S. cerevisiae) (RAD50)
RAD51	RAD51 recombinase (RAD51)
RRM2B	ribonucleotide reductase M2 B (TP53 inducible) (p53R2)
XPA	xeroderma pigmentosum, complementation group A (XPA)
XPC	xeroderma pigmentosum, complementation group C (XPC)
